# Establishment and application of the performance appraisal system for hierarchical diagnosis and treatment in China: A case study in Fujian province

**DOI:** 10.3389/fpubh.2023.1008863

**Published:** 2023-03-02

**Authors:** Qinde Wu, Zijun Zhao, Xianyu Xie

**Affiliations:** ^1^Department of Medical, Fujian Medical University Union Hospital, Fuzhou, Fujian, China; ^2^School of Humanities and Social Sciences, Fuzhou University, Fuzhou, Fujian, China

**Keywords:** hierarchical diagnosis and treatment, performance appraisal system, application research, allocation of resources, health management

## Abstract

**Purpose:**

Hierarchical diagnosis and treatment, as an important measure and direction for China's medical reform, are conducive to improving the capacity of medical services and the national level of health. In this study, a hierarchical diagnosis and treatment performance evaluation index system is established to identify the effects of different influencing factors on developing hierarchical diagnosis and treatment.

**Methods:**

In this study, samples collected from 23 representative integrated medical institutions in nine Fujian cities from 2018 to 2020 are taken as subjects. A hierarchical diagnosis and treatment performance appraisal system is established based on the mechanism of research on the operation of hierarchical diagnosis and treatment. This is combined with the evaluation index system established by the Health Development Research Center, the National Health Commission of the People's Republic of China for the evaluation of hierarchical diagnosis and treatment and the construction of the medical treatment alliance, including studies carried out by related scholars. The weight of each evaluation index is determined with the CRITIC method, and the hierarchical diagnosis and treatment effects on 23 subjects are quantitatively evaluated by the Gray correlation method based on the weight of each index.

**Results:**

The hierarchical diagnosis and treatment performance evaluation index system is established from three aspects, namely allocation of hierarchical diagnosis and treatment resources, establishment of the hierarchical diagnosis and treatment management system, and hierarchical diagnosis and treatment implementation effect; 27 tertiary indexes are formed in total. The Gray correlation of each year in Fujian exceeds 0.5, but <0.53.

**Conclusion:**

Gray correlation of each year in Fujian has gradually increased. But there is still room for improvement. The government departments must improve the investment in medical resources with measures adjusted according to local conditions, promote a balanced allocation of resources for hierarchical diagnosis and treatment, increase communication and interaction between upper and lower medical institutions, and optimize the allocation of resources for hierarchical diagnosis and treatment. Then determine the types of disease to be treated, expand the coverage of chronic disease management, establish standardized chronic disease health management, and strengthen training of health management staff.

## 1. Introduction

With an aging population and the aggravation of chronic noninfectious diseases in China, the inequitable distribution structure of health resources, and the problem of difficult and expensive medical treatment, China presents the hierarchical diagnosis and treatment system, which refers to classification according to the severity of the disease and difficulty of treatment. Different levels of medical institutions bear the treatment of different diseases, and gradually realize the medical process from general practice to specialization and “primary diagnosis, two-way referral, acute and chronic treatment, upper and lower linkage” (1–4). At the end of 2017, there were 321 pilot cities for hierarchical diagnosis and treatment in China, accounting for 95% of cities above the prefecture level. However, according to the China Health Statistical Yearbook, in 2018, there were 2,548 tertiary hospitals in China, accounting for 7.7% of the total number of hospitals. Medical visits from tertiary hospitals account for 51.7% of the total number, and the number of inpatients from tertiary hospitals accounts for 46.4%, while only 6.1 and 6.1% for primary hospitals account for 32.8% of the total number of hospitals (5). Data shows that although the hierarchical diagnosis and treatment system has been implemented throughout the country, there are still “inverted triangle” medical flows, unreasonable distribution and use of health resources, insufficient service capacity of primary medical institutions, insufficient motivation for the implementation and many obstacles in the implementation process.

In view of this, it is of great importance to further evaluate the hierarchical diagnosis and treatment system in China. This study will summarize the important factors that affect the effectiveness and sustainable development of this system in China, establish an evaluation methodology, provide a decision-making reference for the scientific promotion and acceleration of the system, and provide basic support for the realization of the strategic goal of healthy China.

### 1.1. Review of the literature

Several scholars have conducted empirical research on hierarchical diagnosis and treatment. Wang (6) conducted an evaluation and analysis on the construction conditions of the hierarchical diagnosis and treatment system in Jiangsu province based on synergy theory from aspects of multi-departmental policy, e hierarchical health service system synergy, and the medical and health service synergy from the perspective of cognitive evaluation; in which the analysis of policy synergy involves the analysis of policy development and implementation. The synergy of the hierarchical health service system involves the analysis of the synergy of the allocation of health resources and the business of medical institutions, while the medical and health service synergy involves the cognitive evaluation of medical staff and patients on the hierarchical diagnosis and treatment system Its influencing factors include age, education background, work status, average monthly income, place of residence, availability of space, history of any major disease, and awareness of the grass-root first diagnosis system. Zhou (7) based on the theoretical framework of the “result chain” model, evaluated the effects of urban grass-root medical institutions before and after the implementation of hierarchical diagnosis and treatment, based on indices such as policy input, input of medical health resources, fund investment, improvement of primary service capabilities, participation in the construction of the Medical Treatment Alliance, telemedicine, first grass-root diagnosis and treatment and treatment and management rate of chronically ill patients, and their cognition and satisfaction. Lin (8) compared and analyzed the realization of hierarchical diagnosis and treatment before and after the implementation of different models of the medical alliance in Wuhan by stakeholder analysis, based on the indexes of developing human resources in the member units of the Medical Treatment Alliance, the outpatient ratio between the member units and the main hospitals of the Alliance, as well as developing service capabilities, developing service efficiency, and developing financial conditions of such member units. Chen et al. (9) suggested that the effect of hierarchical diagnosis and treatment can be evaluated based on the theoretical framework of “structure-process-result”. Bai et al. (10) established a method for evaluating the expected effect of hierarchical diagnosis and treatment involving outpatient triage, rehabilitation triage, and long-term care triage based on expert consultation and data simulation, and also performed an empirical simulation of the expected effect of triage based on the database of outpatient and hospitalized patients in a certain region in East China in 2016. Yao et al. (11) based on descriptive analysis, evaluated the effects of the hierarchical pilot diagnosis and treatment in Huining county, Gansu province from the aspects of medical services, medical costs of patients, operation of the NCMS medical healthcare insurance fund, income of medical and health institutions, two-way referral and multi-site practice of physicians. Yang et al. (12) based on stakeholder theory evaluated the satisfaction of medical institutions and medical staff (the suppliers), residents/patients (the demanders), government departments (the organizers), and medical enterprises with the effects before and after the implementation of hierarchical diagnosis and treatment. Jia et al. (13) analyzed and compared the hierarchical diagnosis and treatment practice modes in Jurong City of Jiangsu Province, Yicheng City of Hubei Province and Jiulongpo District of Chongqing City from the aspects of top-level design, use of grassroots medical resources, and control of medical costs. Qin et al. (14) with City A in Jiangsu province as an example, analyzed the current state of implementation of the hierarchical diagnosis and treatment system, involving the hierarchical allocation of health resources and medical services. Yang et al. (15) performed a quasi-natural experiment based on the pilot implementation of the hierarchical diagnosis and treatment system in Chongqing City in 2008, and investigated whether the hierarchical diagnosis and treatment system can help alleviate the “difficulty of receiving medical treatment at a low cost” and achieve “medical treatment for all” based on variables related to patients, medical institutions, and basic medical insurance for urban workers. Wang et al. (16) pointed out that the preliminary evaluation of the effects of hierarchical diagnosis and treatment in a certain region can be performed from the aspects of first diagnosis of the root, referral in two ways, treatment tendency, quality and quantity of services, cost and patient experience. Shi et al. (17) evaluated government-run Chinese medicine hospitals in China using ultra-efficient DEA from the perspective of scale income of Chinese medicine hospitals, involving the indexes of the number of medical institutions and the number of beds. Zhang et al. (18) quantitatively evaluated the effectiveness of policies regarding the reform of hierarchical diagnosis and treatment in Xiamen City by the double-difference method, with grade III-A hospitals in Xiamen as the experimental group and those in Fujian, Guangdong, and Jiangsu as the control group. Tao et al. (19) compared the effects in 2 years before and after implementation of hierarchical diagnosis and treatment, and evaluated the effect of hierarchical diagnosis and treatment in Chaoyang district based on volume of medical services, volume of referral in two ways, communication between medical personnel and score of basic medical service indexes.

In 2000, WHO proposed the basic theory and evaluation system of health system performance evaluation in *The World Health Report 2000: Health Systems: Improving performance*, which provided an important reference for evaluating the performance of health services in countries around the world. With the goal of realizing Good Health, Responsiveness and Fairness of Financing, the evaluation was performed based on the active life expectancy, health equity index, responsiveness level index, responsiveness equity index and financing equity index. The evaluation framework indexes proposed by Organization for Economic Co-operation and Development (OECD) countries mainly include: health need, quality (effectiveness, safety, responsiveness, and accessibility), access, cost and expenditure, efficiency, and equity; in addition to health system performance, health status, nonmedical influencing factors, health system design, and background were also integrated. International Organization for Standardization (ISO) health system performance evaluation mainly involves the indexes such as acceptability, accessibility, safety, efficiency, competence, effect, and continuity, as well as the evaluation dimensions of community and health systems, nonmedical influencing factors, and health status. The National Institute for Health and Care Excellence (NICE), proposed a key index system of the health system performance evaluation framework, which believed that public health would be affected by multiple influencing factors, including population, organization, environment, and society. In the 1980s, American Public Health Association determined the connotation of health service performance evaluation as a process of judging the number, progress, and value of the predetermined goals, involving the following five aspects: (1) Determination of health goals: The evaluation indices should clearly reflect the goals of health work, and should be used for evaluating the realization of these goals; therefore, the basic premise of evaluation is to determine the health goals; (2) Clarification of the progress of the goals, namely the health service evaluation, should clarify the realization of health service goals; (3) Measurement and judgment of the goal realization effect, which is the basic function of health service evaluation; (4) Measurement of the social and economic benefits achieved by realization of the goals: Health service evaluation must be able to measure social and economic benefits achieved by the realization of the goals; (5) recommendations for future work through the evaluation of health service evaluation; using Quality Outcomes Framework (QOF), involving clinical, institutional, and ancillary services and patient experience, as well as the salary level of general practitioners. WHO designed an evaluation and supervision framework for medical and health system reform in China, including four progressive stages. Input and process-output-outcome-impact, in which input and process involve governance, financing, infrastructure, health workforce, supply chain, and information; output involves the access to interventions, willingness to serve, and quality, safety, and efficiency of interventions; outcome involves coverage of interventions, risk of diseases, behaviors, and risk factors; and impact involves the improvement of health outcomes and equity, and the protection from social and financial risks.

Currently, scholars attach greater importance to the measurement of financial indexes and the quality of medical services, which is not applicable to the connotation, characteristics, and objectives of hierarchical diagnosis and treatment in China. Musgrove (20) believed that it was unreasonable to apply the same evaluation indexes to all countries, considering the difference in national conditions and historical development stages. Therefore, all countries should establish evaluation index systems that are in accordance with their own national conditions. Currently, Chinese scholars generally focus on simple descriptive analysis, or the effectiveness of hierarchical diagnosis and treatment, which cannot reflect the problems and deficiencies in the operation of hierarchical diagnosis and treatment.

Current studies lack systematic evaluation of the performance of the hierarchical diagnosis and treatment system and cannot discover all the problems. Therefore, this study will establish a hierarchical diagnosis and treatment performance appraisal system from the source, process, and results of the hierarchical diagnosis and treatment system in combination with its nature and characteristics, so that it can evaluate the performance of the hierarchical diagnosis and treatment system more comprehensively.

## 2. Proposed methodology

### 2.1. Establishment of the performance evaluation index system for hierarchical diagnosis and treatment

The key process to establish an evaluation system is to select the evaluation indexes. In this study, based on studies on the mechanism of operation of hierarchical diagnosis and treatment, and in combination with the evaluation index system established by the Center for Research on Health Development and the National Health Commission of the People's Republic of China for the evaluation of hierarchical diagnosis and treatment and the establishment of the Medical Treatment Alliance, as well as related studies, a hierarchical diagnosis and treatment performance evaluation index system is established from the aspects of allocation of hierarchical diagnosis and treatment resources, establishment of the hierarchical diagnosis and treatment management system, and the effect of hierarchical diagnosis and treatment implementation effect. There are 27 tertiary indexes in total, which can be taken as important references and evaluation basis for evaluating the performance of hierarchical diagnosis and treatment, including 10 resource allocation indexes, 6 system establishment indexes, and 11 implementation effect indexes.

#### 2.1.1. Allocation of hierarchical diagnosis and treatment resources

The allocation of hierarchical diagnosis and treatment resources refers to the input for the implementation of hierarchical diagnosis and treatment. The input stage involves building the capacity of the service and the interaction of medical institutions. Evaluation indexes are determined from two aspects:

##### 2.1.1.1. Building capacity for the service of medical institutions

The building of service capacity of medical institutions involves the infrastructure of grassroots medical institutions and the high-quality development of public hospitals. The infrastructure of grassroot medical institutions covers the building of personnel teams, the financial input and the input of material resources. The building of personnel teams means that the grassroots medical institutions must establish their own medical service teams with professional proficiency and adequate staff, and hospitals must have high-quality medical talents with high academic qualifications and high technical capabilities. The proportion of (assistant) physicians assigned by hospitals to primary health and medical institutions reflects the importance that superior hospitals attach to subordinate institutions. This is an important measure to improve the capacity of the basic medical service and attract patients for first diagnosis (21). In the Outline of the National Medical and Health Service System Plan (2015–2020), the number of practicing physicians per 1,000 permanent residents was considered one of the major indexes for the allocation of resources in the national medical health service system. As required in the outline, the number of practicing physicians (assistant) physicians per 1,000 permanent residents should reach 2.5 in 2020. The allocation of quantity of grassroots physicians is an important symbol that reflects the rational distribution of medical and health personnel in urban and rural areas, which is a resource allocated according to the health service needs of the people in China (22). Since financial resources are part of medical resources, as for hierarchical diagnosis and treatment, the government should focus on special input in hierarchical diagnosis and treatment. The gap between grass-root medical institutions and superior hospitals is mainly caused by the obvious advantages of superior hospitals in financial support and capital strength. Therefore, the government should further strengthen the financial support for grass-root medical institutions, such as the financial support for the construction of basic public health services, chronic disease management, family doctor contracting service, and electronic file informatization. Furthermore, the special fund to subsidize the loss of medical institutions caused by hierarchical diagnosis and treatment can stimulate the improvement of grassroots medical and health services, encourage superior hospitals to dispatch physicians to support grassroots medical institutions, and also encourage them to carry out scientific research and establish medical centers. Financial special funds for hierarchical diagnosis and treatment construction per 1,000 permanent residents represent the government's continuous attention and fund input regarding hierarchical diagnosis and treatment (23). The input of material resources mainly involves the input of software facilities and hardware facilities; grass-root medical institutions should be attached with a certain number of advanced medical equipment and facilities. In the primary health institutions of China, there are rare facilities with a unit price of more than 1 million yuan, which can only be used for the diagnosis and treatment of basic diseases. The proportion of facilities with a unit price of more than 1 million yuan in all facilities is the concentrated expression of the levels of medical services in grass-root medical institutions, and also an important index for evaluating the performance of hierarchical diagnosis and treatment (24). In the Outline of the National Medical and Health Service System Plan (2015–2020), it was required that the number of beds per 1,000 permanent residents in primary medical and health institutions reach 1.2 in 2020, and the number of beds is an important index for meeting the needs of patients in hospitals. Therefore, the number of beds per 1,000 permanent residents in primary medical and health institutions is crucial for “grassroot first diagnosis and two-way referral” required by hierarchical diagnosis and treatment.

Personnel serves as the core of high-quality development of public hospitals, hierarchical diagnosis and treatment requires “grass-root first diagnosis, and two-way referral”; the superior and subordinate medical institutions should perform labor division and cooperation; in particular, grass-root medical institutions should be responsible for the treatment of chronic diseases and common diseases, while superior hospitals should be responsible for the treatment of complex acute and critical diseases. Superior hospitals should pay attention to the development and application of high- and new medical technologies, scientific research, and diagnosis and treatment of acute and critical diseases. Therefore, hospitals should attract high-quality and high-tech medical talents and seek more scientific research funds to support medical scientific research, thus forming a virtuous circle. The indexes that can be easily measured include the proportion of MD or PhD of hospital health technicians and scientific study funds per 100 health technicians in hospitals. Medical staff with master's and doctoral degrees can rapidly improve medical, teaching and research capabilities, and the overall strength of hospitals (25); scientific research fund, as the guarantee of funding for medical scientific research, can continuously stimulate and promote the development of medical research. Therefore, seven indexes are selected for measurement in this paper, namely proportion of (assistant) physicians assigned by hospitals to the primary medical and health institutions, number of (assistant) physicians per 1,000 permanent residents in primary medical and health institutions, number of equipment above 10,000 yuan per 1,000 permanent residents in primary medical and health institutions, number of beds per 1,000 permanent residents in primary medical and health institutions, financial special funds for hierarchical diagnosis and treatment construction per 1,000 permanent residents, and proportion of MD or PhD of hospital health technicians.

##### 2.1.1.2. Interaction

Hospitals can improve service capabilities by sinking resources and driving radiation. Hierarchical diagnosis and treatment should focus on strengthening grass-root medical institutions; It is not enough to rely on grass-root medical institutions to improve service capabilities; grass-root medical personnel should be sent to superior hospitals to learn and improve their technical levels under professional technical guidance and teaching from health technicians in superior hospitals, thus promoting the improvement of the professional and technical levels of grass-root health technicians and the capacity for medical service of grass-root medical institutions, and completing new tasks that combine prevention and treatment in grass-root medical institutions under the strategy of Healthy China (26). The learning of health technicians at the grassroots level in hospitals is a necessary path of further training and improvement, which can be measured by the index “number of people per 100 health technicians at the primary institutions studying in hospitals”. The construction of grass-root medical institutions depends on the assistance of and interaction with superior hospitals, as well as the driving by interdisciplinary talents in superior hospitals. An important measure to consolidate the medical resources of grass-root medical institutions is to regularly send medical experts to grass-root medical institutions to provide guidance, and the index “number of people per 100 health technicians in the hospitals to the primary medical institutions for guidance” can illustrate the continuous guidance and support of grass-root medical institutions by superior hospitals, which can play a crucial role in the rapid improvement of the medical service of grass-root medical institutions. The number of new technologies and new projects is important for developing grass-root medical institutions; In the past, the weak medical service capacity of grass-root medical institutions was denounced by the public, which was also the main reason for the failure to form “grass-root first diagnosis and referral 2.” Most people would not seek a first diagnosis in grass-root medical institutions, therefore, improving the diagnosis and treatment level of grass-root medical institutions is a basic requirement for hierarchical diagnosis and treatment. The number of new technologies and projects is a sign of measuring the improvement of the level of diagnosis and treatment of grass-root medical institutions, and the higher number of new technologies and new projects performed by hospitals and primary health technicians per 100 can continue to promote “grass-root first diagnosis, and two-way referral” (27). Therefore, three indices are selected for measurement, namely the number of people per 100 health technicians at the primary institutions studying in hospitals, the number of people per 100 health technicians in the hospitals to the primary medical institutions for guidance, and the number of new technologies and new projects performed by hospitals and primary health technicians per 100.

#### 2.1.2. Establishment of the hierarchical diagnose and treatment management system

The establishment of the hierarchical diagnosis and treatment management system is a process of management of hierarchical diagnosis and treatment. The stages of hierarchical diagnosis and treatment management include “the establishment of the incentive system” and “the establishment of the division and cooperation system”; therefore the evaluation indices will be determined from the following two aspects:

##### 2.1.2.1. Establishment of the incentive system

Implementing the hierarchical diagnosis and treatment system involves the behaviors of three stakeholder groups, including the relinquishment of the hospitals, the acceptance by the grass-root medical institutions, and the willingness of the patients to seek medical treatment in grass-root medical institutions. With the incentive compatibility method, the behaviors of the actors must be consistent with the collective goal. The preparation and implementation of public policies should be completed based on the financial policies of the government, for the government can promote medical institutions to participate in hierarchical diagnosis and treatment and promote superior and subordinate medical institutions to participate in labor division and cooperation; improve integration and coordination between superior and subordinate medical institutions; increase income and benefits of medical staff; make medical institutions return to public welfare; and promote two-way referral, especially referral of patients at the stage of rehabilitation and those with common diseases to grass-root medical institutions, thus gradually forming a treatment order of “grass-root first diagnosis, and two-way referral” (28). Medical insurance incentive system is important for the development of hierarchical diagnosis and treatment; while medical insurance, as an important measure for new medical reform to promote hierarchical diagnosis and treatment and also the main payer of medical expenses in China, plays a key role in the medical industry and a powerful tool to regulate the behaviors of both physicians and patients; also it can also regulate orderly medical treatment of patients, regulate medical institutions to reasonably perform diagnosis and treatment, reduce medical costs and save medical expenses; therefore, it is an important guarantee for the formation of the order of “grass-root first diagnosis, and two-way referral” (29). The premise for hierarchical diagnosis and treatment is to establish a hierarchical diagnosis and treatment management system, including the establishment of the work quality assessment system for medical institutions. The quality of medical institutions' work, as a concentrated reflection of service capabilities, is the lifeline of medical institutions, a key index for medical treatment and also a key aspect for the evaluation of medical services. The establishment of the quality assessment system for medical institutions is the key to building the capacity of medical institutions to provide services, which plays an important role in hierarchical diagnosis and treatment and serves as an important index of the performance of hierarchical diagnosis and treatment (30). As the most important content in medical reform policies, hierarchical diagnosis and treatment involves multiple links and contents in the health system such as assessment and distribution of personnel and salary, from which it can be concluded that the establishment of the hierarchical diagnosis and treatment system is a systematic project. Due to the strong specialty of medical and health services, it is necessary to perform a careful division of labor in medical and health institutions at all levels when establishing the hierarchical diagnosis and treatment system; at the same time, medical and health institutions at all levels should engage in cooperation. Therefore, it is necessary to establish a good HR and remuneration system and establish an incentive mechanism (31). Therefore, the government financial investment policy, medical insurance incentive system, medical institution work quality assessment system and medical institution HR, and remuneration system are used to evaluate the establishment of the hierarchical diagnosis and treatment management system.

##### 2.1.2.2. Establishment of the labor division and cooperation system

Labor division and cooperation involve the determination of the content of the division of labor and the establishment of cooperative relations, as well as the establishment of the entire service system for the referral of patients between hospitals and grassroots medical institutions and hospitals. Mean while, the obstacles found in the referral process should be unified and coordinated. The reform of hierarchical diagnosis and treatment is a systematic project covering political, economic, social, health, and even cultural fields and involving multiple links and contents in the health system such as medical insurance, price, HR, and remuneration assessment and distribution. To achieve the high-quality development of hierarchical diagnosis and treatment, various resources should be integrated, especially the integration and optimization of medical resources. The establishment of the resource integration system is a prerequisite to clarify the way of integrating medical resources, optimizing the allocation of medical resources, and preventing the sinking of high-quality medical resources (32). Hierarchical diagnosis and treatment should focus on the medical treatment order of “grassroot first diagnosis, and hierarchical referral”; labor division and cooperation between medical institutions at all levels should be implemented throughout hierarchical diagnosis and treatment; The premise of labor division and cooperation is the establishment of the system of division of resources. Due to the strong specialty of medical and health services, a detailed division of labor must be performed in medical and health institutions at all levels when establishing the hierarchical diagnosis and treatment system. Meanwhile, medical and health institutions at all levels should cooperate. Therefore, a rational resource division system must be established to support the institutional environment and promote the establishment of the hierarchical diagnosis and treatment management system (33). In this paper, the resource integration system and the resource division system are selected for evaluation.

#### 2.1.3. Hierarchical diagnosis and treatment implementation effect

The implementation effect of hierarchical diagnosis and treatment is the transformation and feedback link during the implementation of hierarchical diagnosis and treatment. The transformation and feedback of hierarchical diagnosis and treatment cover the direct effect and long-term benefit, involving triage effect, health benefit, and medical cost control. Evaluation indexes are determined from the following three aspects:

##### 2.1.3.1. Triage effect

As for the most intuitive effect, the triage effect can promote the sinking of high quality resources and improve the capacity for medical services of grassroots medical institutions through the interaction and coordination of medical personnel between grassroots medical institutions and superior hospitals (34). The proportion of outpatient and emergency visits in primary care facilities is the most intuitive index of hierarchical diagnosis and treatment. Its improvement can help improve service efficiency, reduce waste of resources, and promote the coordinated development of medical institutions. Use of hospital beds refers to the ratio of daily occupied beds to owned beds, i.e., the ratio obtained by dividing the actual occupied beds by accessible beds. Currently, the utilization of hospital beds is relatively low in grassroots medical institutions in China, and on the contrary, beds in superior hospitals are always fully occupied. According to the ideal state of hierarchical diagnosis and treatment, the use of hospital beds in primary care institutions should be at a relatively high level, to play the role of shunting and alleviating the “difficulty of getting medical services” from large hospitals. The use of hospital beds in local medical institutions is an intuitive reflection of the realization of hierarchical diagnosis and treatment (35). Hierarchical diagnosis and treatment require “first national diagnosis and two-way referral”; the superior and subordinate medical institutions must perform labor division and cooperation; specifically, grassroots medical institutions must be responsible for the treatment of chronic diseases and common diseases, while superior hospitals must be responsible for the treatment of complex acute and critical diseases. Regarding superior hospitals, one of the most intuitive features of the treatment of complex acute and critical diseases is the proportion of surgeries of level 3/4 or Class C/D surgeries, which also reflects the comprehensive strength of hospitals and the guarantee of medical visits at integrated medical institutions (36). As required by hierarchical diagnosis and treatment, superior hospitals must be responsible for the treatment of complex acute and critical diseases and must also improve the level of medical diagnosis and treatment. In addition to the proportion of Level 3/4 surgeries or Class C/D surgeries, the coincidence rate of the main diagnosis upon admission and discharge is also a concentrated expression of the general strength of hospitals and the guarantee of medical visits at integrated medical institutions (37). Hierarchical diagnosis and treatment aim to provide patients with continuous and accessible medical services. Accessibility means that patients can enjoy the same basic medical services as those in hospitals at the grass-root medical institutions. Continuity means that patients can enjoy uninterrupted medical services throughout the life cycle through information sharing and cooperation between medical institutions. Referral is an important requirement for hierarchical diagnosis and treatment, and also a major path to ensure that patients can enjoy continuous, accessible medical services. The proportion of referrals is an intuitive index that refutes the realization of hierarchical diagnosis and treatment (38). Therefore, the proportion of outpatient and emergency visits in grass-root medical institutions, the utilization of hospital beds in grass-root medical institutions, the proportion of Level 3/4 surgeries or Class C/D surgeries, the coincidence rate of the main diagnosis upon admission and discharge and the proportion of referrals are used for evaluation.

##### 2.1.3.2. Health benefits

Under the background of Healthy China, the goal of hierarchical diagnosis and treatment is to improve the health level. Based on the theory of public policy, social benefits refer to the benefits of improving people's social living standards by policy reform (39). Medical and health system reform, belonging to the category of public policies, aims to improve the health of the public, and also make the people treated suffer from fewer diseases, and affordable, which is the reflection of social benefits. Therefore, the main criterion for evaluation is to improve the health of the public and reduce the economic burden of patients. Infant mortality is an important index measuring the health and medical level of a region. Hierarchical diagnosis and treatment should focus on strengthening grassroots medical institutions and asking them to concentrate various medical resources to manage chronic diseases and health, thus improving the public's health. Regarding the specific effect, the hierarchical diagnosis and treatment system can cause people to suffer from “fewer diseases” (40). Chronic diseases are the diseases with the highest proportion. The chronic disease management rate in a region can directly improve the health level and can be taken as the main index for evaluating the regional medical level and hierarchical diagnosis and treatment level. Family physicians, also known as general physicians, provide medical care services primarily to families. In China, the family physician team develops slowly and has not played a role in the management of chronic disease management. Therefore, it is necessary to improve the coverage of family physicians, which is a major evaluation index to ensure convenience of medical treatment, improve the health level in each region, and promote the development of hierarchical diagnosis and treatment (41). The infant mortality, chronic disease management rate, and coverage of family physicians are selected for evaluation.

##### 2.1.3.3. Medical cost control

The hierarchical diagnosis and treatment mainly intend to control medical costs, reduce medical expenses, reduce the cost, and alleviate the burden of medical treatment for patients through division of labor and referral between superior and subordinate medical institutions. Regarding the control of medical costs, the hierarchical diagnosis and treatment system can reasonably drive patients, prevent the waste of medical resources, and effectively control the medical cost, thus containing the increase in medical costs. Compared to outpatient emergency treatment, the proportion of hospitalization cost is quite high; therefore, the rate of change of self-paid hospitalization cost is selected as a representative index to measure medical cost and medical burden (42). The balance of the medical insurance fund refers to the difference between the income and expenditure of the medical insurance fund. The medical insurance fund, as the main payer for medical care currently, is a major source of medical income. Its balance can be taken as the capital base for the continuous development of medical institutions and improvement of the comprehensive strength; while the balance rate is a key index for evaluating cost control and internal management of medical institutions (43). Through adjustment of service prices, hierarchical diagnosis and treatment can allow medical institutions to realize the balance of financial revenue and expenditure and obtain economic benefits, thus stimulating them to promote the smooth operation of hierarchical diagnosis and treatment. To guarantee the continuous operation of the hierarchical diagnosis and treatment system, medical institutions must engage in a long-term and effective operation; The annual balance can realize a virtuous circle of sustainable development and improvement of the level of medical service, and it is a basic condition to realize hierarchical diagnosis and treatment (44). Therefore, the change rate of self-funded hospitalization cost, the balance rate of the medical insurance fund and the annual balance rate are selected for evaluation.

In summary, a performance evaluation index system for hierarchical diagnosis and treatment in Fujian is established, as shown in Table 1.

**Table 1:** Performance evaluation indicator system of the hierarchical diagnosis and treatment system.

**Level 2 indicators**	**Level 3 indicators**
Service capacity building of medical institutions A	proportion of (assistant) physicians assigned by hospitals to primary health and medical institutions (A_1_)
	number of (assistant) physicians per 1,000 permanent residents in primary health and medical institutions (A_2_)
	number of equipment >10,000 yuan per 1,000 permanent residents in primary medical and health institutions (A_3_)
	number of beds per 1,000 permanent residents in primary medical and health institutions (A_4_)
	Financial special funds for hierarchical diagnosis and treatment construction per 1,000 permanent residents (A_5_)
	proportion of MD or Ph.D. of hospital health technicians (A_6_)
	Scientific study funds per 100 health technicians in hospitals (A_7_)
Communication and interaction B	number of people per 100 health technicians in primary institutions studying in hospitals (B_1_)
	number of people per 100 health technicians in hospitals to primary medical institutions for guidance (B_2_)
	number of new technologies and new projects carried out by hospitals and primary health technicians per 100 (B_3_)
Incentive compatibility C	government financial investment policy (C_1_)
	medical insurance incentive system (C_2_)
	Work quality assessment system of medical institutions (C_3_)
	Personnel salary system of medical institutions (C_4_)
Division of labor and cooperation D	Resource integration system (D_1_)
	Resource division system (D_2_)
Triage effect E	proportion of outpatient and emergency visits to primary medical and health institutions (E_1_)
	Rate of utilization of inpatient beds in primary medical and health institutions (E_2_)
	Proportion of level III and IV surgery in the hospital (E_3_)
	Coincidence rate of main diagnosis on admission and discharge on hospital (E_4_)
	Proportion of Up-down Referrals (E_5_)
Health benefits F	infant mortality (F_1_)
	Chronic disease management rate (F_2_)
	Contracted coverage rate of family physicians (F_3_)
Medical cost control G	Change in the rate of self-payment burden per hospitalization (G_1_)
	Balance rate of the medical insurance fund (G_2_)
	Annual balance rate of revenue and expenditure (G_3_)

### 2.2. Determination of index weight based on the CRITIC method

#### 2.2.1. Processing of data normalization

Data standardization: the range transformation method is used to process the data, so that the positive and reverse indicators are converted into positive indicators and the values are between 0 and 1. The optimal value is 1, and the worst value is 0.

For positive indicators:


(1) $$\begin{array}{l} y_{ij}=\frac{x_{ij}-\min_{i}\left(x_{ij}\right)}{\max_{i}\left(x_{ij}\right)-\min_{i}\left(x_{ij}\right)} \end{array}$$

For reverse indicators:


(2) $$\begin{array}{l} y_{ij}=\frac{\max_{i}\left(x_{ij}\right)-x_{ij}}{\max_{i}\left(x_{ij}\right)-\min_{i}\left(x_{ij}\right)} \end{array}$$

In the formula, *x*_*ij*_ represents the value of the item *j* for the *i* sample, *y*_*ij*_ represents numerical value after dimensionless treatment, *i*=1,⋯, *n*,j=1,⋯, *p*.

#### 2.2.2. Calculation of weight value

Determining indicator weight: The CRITIC method comprehensively considers the influence of indicator variation on weight and the conflict between indicators: the standard deviation reflects the indicator contrast strength and the correlation coefficient reflects the degree of indicator conflict.


$$\begin{array}{l} C_{j}=\sigma_{j}{\sum^{\text{​}}}_{k=1}^{p}(1-r_{jk}).\\ \;w_{j}=\frac{C_{j}}{{\sum^{\text{​}}}_{j=1}^{p}C_{j}}. \end{array}$$

In the formula, **C**_**j**_ represents the impact of the **j** evaluation indicator on the system; σ_**j**_ is the standard deviation of the evaluation indicator**j**,indicates the contrast strength between the indicator **j** and other indicators; **r**_**jk**_ is the correlation coefficient between indicators **j** and **k**, $\sum_{k=1}^{p}\left(1-r_{jk}\right)$ indicates the conflict between the indicator **j** and other indicators. Therefore, the greater the **C**_**j**_,means it contains more information, the greater the weight. In the above, **w**_**j**_ represents the objective weight of the indicator **j** (45).

#### 2.2.3. Comprehensive score calculation

Based on calculating the above weights, the comprehensive scores of each sample can be obtained by weighted average of the normalized data. The formula is as follows:


$$\begin{array}{l} I_{i}=\sum_{j=1}^{p}w_{j}y_{ij}. \end{array}$$

According to the above CRITIC calculation method, the weight of the indicators system can be calculated as in Table 2.

**Table 2:** Weight value and ranking of the performance evaluation indicator system of the hierarchical diagnosis and treatment system.

	**Level 3 indicators**	**Weight**	**Ranking**
Service capacity building of medical institutions A	Proportion of (assistant) doctors assigned by hospitals to the primary medical and health institutions (A_1_)	0.0272	26
	Number of (assistant) doctors per 1,000 permanent residents in primary medical and health institutions (A_2_)	0.0387	12
	Number of equipment >10,000 yuan per 1,000 permanent residents in primary medical and health institutions (A_3_)	0.0471	2
	Number of beds per 1,000 permanent residents in primary medical and health institutions (A_4_)	0.0335	19
	Financial special funds for hierarchical diagnosis and treatment construction per 1,000 permanent residents (A_5_)	0.0361	17
	Proportion of MD or PhD of hospital health technicians (A_6_)	0.0366	16
	Scientific study funds per 100 health technicians in the hospitals (A_7_)	0.0422	8
Communication and interaction B	Number of people per 100 health technicians in primary institutions studying in hospitals (B_1_)	0.0320	21
	Number of people per 100 health technicians in hospitals to primary medical institutions for guidance (B_2_)	0.0398	10
	Number of new technologies and new projects performed by hospitals and primary health technicians per 100 (B_3_)	0.0274	25
Incentive compatibility C	Government financial investment policy (C_1_)	0.0430	6
	Medical insurance incentive system (C_2_)	0.0422	9
	Work quality assessment system of medical institutions (C_3_)	0.0339	18
	Personnel salary system of medical institutions (C_4_)	0.0442	5
Division of labor and cooperation D	Resource integration system (D_1_)	0.0257	27
	Resource division system (D_2_)	0.0454	3
Triage effect E	Proportion of outpatient and emergency visits to primary medical and health institutions (E_1_)	0.0429	7
	Utilization rate of inpatient beds in primary medical and health institutions (E_2_)	0.0478	1
	Proportion of level III and IV surgery in the hospital (E_3_)	0.0369	14
	Coincidence rate of main diagnosis on admission and discharge from hospital (E_4_)	0.0323	20
	Proportion of up-down referrals (E_5_)	0.0381	13
Health benefits F	Infant mortality (F_1_)	0.0369	15
	Chronic disease management rate (F_2_)	0.0389	11
	Contracted coverage rate of family doctors (F_3_)	0.0449	4
Medical cost control G	Change in the rate of self-payment burden per hospitalization (G_1_)	0.0297	22
	Balance rate of the medical insurance fund (G_2_)	0.0280	24
	Annual balance rate of revenue and expenditure (G_3_)	0.0287	2

### 2.3. Stability test

To verify the stability of the hierarchical diagnosis and treatment performance evaluation index system, the weight of the sample data is calculated using the CRITIC method and the entropy weight method, and the evaluation scores for each sample are calculated. The rank of the total scores of the two methods is tested using the symbolic rank test.

#### 2.3.1. Entropy weight method

In information statistics, the entropy weight method is usually used as a method to determine the weight. The entropy weight method is an objective weighting method, which can more objectively and truly reflect the influence of variables on the evaluation results. Mainly including the following four steps (46):

(1) Data normalization processing (the same as the steps in “2.2.1”)
(2) Calculation of information entropy based on normalization results.

The formula of specific gravity *p*_*ij*_ under the *j* index of the *i*object is:


$$\begin{array}{l} p_{ij}=y_{ij}/\overset{n}{\underset{i=1}{\sum }}y_{ij}. \end{array}$$

The calculation formula of information entropy *E*_*j*_ of each index is:


$$\begin{array}{l} E_{j}=-\frac{1}{\ln\;n}\overset{n}{\underset{i=1}{\sum }}p_{ij}\ln\;\left(p_{ij}\right). \end{array}$$

If *p*_*ij*_ = 0, then $\underset{p_{ij}=0}{\lim}p_{ij}*\ln(p_{ij})=0_{{}^{\circ}}$

(3) Calculation of each index weight value *w*_*j*_°

$$\begin{array}{l} w_{j}=\frac{1-E_{j}}{\overset{m}{\underset{j=1}{\sum }}\text{1}-E_{j}}. \end{array}$$

(4) Calculation of the composite score of each object_*z*_*i*_°_

$$\begin{array}{l} z_{i}=\overset{m}{\underset{j=1}{\sum }}w_{j}y_{ij}. \end{array}$$

#### 2.3.2. Symbol rank test method

Wilcoxon signed rank test is a nonparametric method for testing whether there are significant differences in the overall distribution of the two paired samples. Assuming that the rankings of each sample are relatively close, the ranking distribution under the two methods should be relatively close. Using this method, we can test whether there are significant differences in the ranking distribution using the two methods. The basic ideas and steps are as follows.

First, the ranking obtained by the CRITIC method of each indicator is subtracted from the ranking obtained by the entropy weight method. According to the absolute value |*d*_*i*_| of the difference between the two methods, it is arranged from small to large and the corresponding rank is given. If the difference item 0 appears, the item is omitted, the rank is not given, and the sample size *n* is reduced accordingly; when the difference is the same, the average rank is taken, and the arithmetic average of rank is used to replace the original rank.

Assuming that the difference values are independent and identically distributed, and they are also symmetrically distributed about point θ, the problem of testing whether there are differences between the two overall distributions can be transformed into the problem of testing whether the symmetric center θ is equal to 0, thus establishing the following original hypothesis:


$$\begin{array}{l} H_{0}\theta =0. \end{array}$$

It is assumed that in the total difference D, the number of positive and negative differences is the same and that they are symmetrically distributed on both sides of the mean 0. There is no difference between total X and total Y. A smaller of the two ranks is usually used as a Wilcoxon T statistic and as a test statistic. Under the premise of the original hypothesis, the mathematical expectations and variance of the T statistics are:


$$\begin{array}{l} E\left(T\right)=\frac{n\left(n+1\right)}{4} \end{array}$$

$$\begin{array}{l} V\left(T\right)=\frac{n\left(n+1\right)\left(2n+1\right)}{24}. \end{array}$$

When *n* ≥ 25 (*n* is the total number of positive and negative numbers, excluding the number of differences 0), T approximately obeys the normal distribution. Statistics Z can be constructed:


$$\begin{array}{l} Z=\frac{T-E\left(T\right)}{\sqrt{V\left(T\right)}}. \end{array}$$

According to the standard normal distribution probability distribution table or using statistical software, the *p*-value or the critical value T can be obtained (47).

#### 2.3.3. Results of the stability test

Based on the existing data, the sign rank sum test is carried out by the Minitab software and the *p*-value is 0.625. There is not enough reason to reject the original hypothesis. Therefore, it can be considered that the weight values calculated by the two methods are not significantly different, so that the evaluation index system is objective and stable, as shown in Table 3.

**Table 3:** Evaluation value and ranking of samples by CRITIC and entropy weight method in Fujian.

**Integrated medical services**	**Evaluation value of entropy weight method**	**Evaluation value of the CRITIC method**	**Ranking of entropy weight**	**Ranking of CRITIC**	**Difference between rankings**
1	0.1748	0.4267	19	11	8
2	0.2520	0.4984	10	5	5
3	0.1386	0.3017	20	22	−2
4	0.3024	0.5271	6	4	2
5	0.2583	0.4260	9	12	−3
6	0.4148	0.5314	3	3	0
7	0.2186	0.4655	13	8	5
8	0.1290	0.3174	22	20	2
9	0.3322	0.4979	4	6	−2
10	0.1876	0.3432	17	18	−1
11	0.2661	0.4055	8	14	−6
12	0.1059	0.2623	23	23	0
13	0.1807	0.3162	18	21	−3
14	0.2392	0.4671	11	7	4
15	0.4576	0.5344	1	2	−1
16	0.4237	0.6235	2	1	1
17	0.2244	0.3714	12	17	−5
18	0.3096	0.4367	5	10	−5
19	0.2016	0.4081	15	13	2
20	0.1384	0.3179	21	19	2
21	0.3017	0.4516	7	9	−2
22	0.1977	0.3720	16	16	0
23	0.2145	0.3934	14	15	−1

### 2.4. Evaluation results

**Figure 1:**
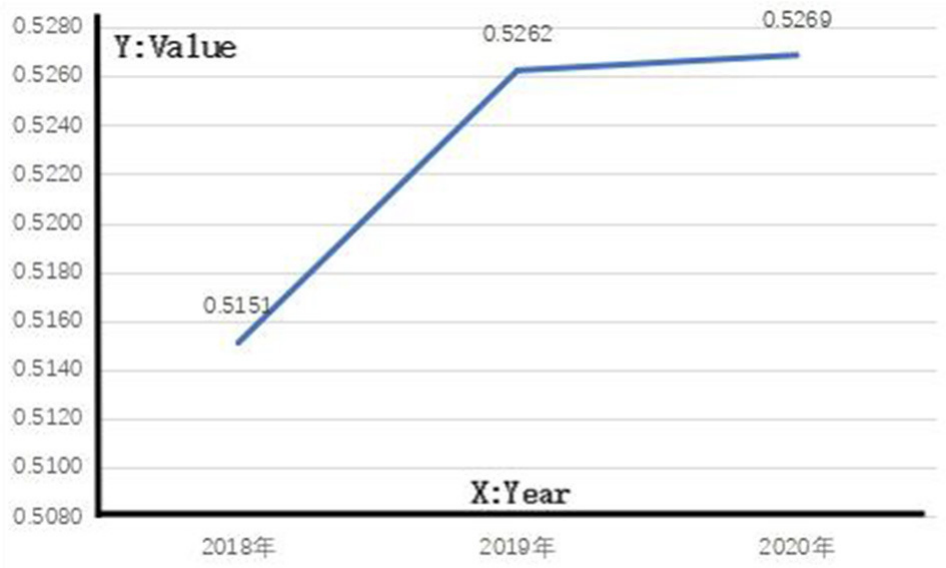
Implementation effect of hierarchical diagnosis and treatment policies in Fujian.

With the gray correlation calculation method, gray correlation values are obtained from 23 integrated medical institutions from 2018 to 2020 and the mean values of such values are used to evaluate the effect of the implementation of hierarchical diagnosis and treatment policies in Fujian. As shown in Figure 1, the mean of gray correlation values of 23 integrated medical institutions in Fujian is above 0.5.

## 3. Analysis and discussion

### 3.1. Analysis on the general conditions of Fujian

As shown in Figure 1, the gray correlation in each year shows an upward trend, indicating the gradual strengthening of the correlation with the reference sequence and the gradual increase of the overall level. The values in 2018, 2019, and 2020 exceeded 0.5, but all were <0.53, indicating that the implementation effects of hierarchical diagnosis and treatment policies in Fujian in the 3 years were at level III, and there was still a lot of room for improvement.

### 3.2. Allocation of hierarchical diagnosis and treatment resources

#### 3.2.1. Service capacity building of medical institutions

The index “service capacity building of medical institutions” consists of “number of (assistant) doctors per 1,000 permanent residents in primary medical and health institutions”, “number of equipment above 10,000 yuan per 1,000 permanent residents in primary medical and health institutions”, “number of beds per 1,000 permanent residents in primary medical and health institutions”, “financial special funds for hierarchical diagnosis and treatment construction per 1,000 permanent residents”, “proportion of (assistant) doctors assigned by hospitals to the primary medical and health institutions”, “proportion of MD or PhD of hospital health technicians” and “scientific study funds per 100 health technicians in the hospitals”, in which, “number of beds per 1,000 permanent residents in primary medical and health institutions” has the strongest impact on the development of hierarchical diagnosis and treatment, followed by “proportion of MD or PhD of hospital health technicians”, “number of equipment above 10,000 yuan per 1,000 permanent residents in primary medical and health institutions”, “number of (assistant) doctors per 1,000 permanent residents in primary medical and health institutions”, “proportion of (assistant) doctors assigned by hospitals to the primary medical and health institutions”, “financial special funds for hierarchical diagnosis and treatment construction per 1,000 permanent residents”, and “scientific study funds per 100 health technicians in the hospitals”, with the weights of 0.0422, 0.0398, 0.0366, 0.0361, 0.0335, 0.0320, and 0.0274, respectively, ranking the eighth, tenth, sixteenth, seventeenth, nineteenth, twenty-first and twenty-fifth in the 27 tertiary indexes. A fundamental way to improve the service capacity of grass-root medical institutions is to increase the input of medical resources in grass-root medical institutions, including human resources, medical equipment, and beds. “number of beds per 1,000 permanent residents in primary medical and health institutions' is the key to providing the capacity of grass-root medical institutions to completely treat common and chronic diseases; “The number of equipment above 10,000 yuan per 1,000 permanent residents in primary medical and health institutions” refers to investment in equipment and facilities for medical institutions to provide medical services; and the most important index in human resources is the number of practicing (assistant) physicians, all of which are important indexes to evaluate the service capacity of grass-root medical institutions, and can attract local residents. “proportion of MD or PhD of hospital health technicians” reflects the medical, teaching and research capabilities and the comprehensive strength of medical institutions; the hierarchical diagnosis and treatment system requires that superior and subordinate medical institutions perform labor division and cooperation, in particular, superior hospitals should be responsible for the treatment of complex acute and critical diseases, and focus on scientific research and the diagnosis and treatment of critical diseases; therefore, it is particularly important to employ highly educated, high-quality and high-tech medical talents.

In recent years, Fujian province has attached more attention to equipment, beds, number of (assistant) physicians and financial investment in grassroots medical institutions, with obvious policy inclination. Significant progress has been made in the equipment, beds, number of (assistant) physicians and financial investment in grass-root medical institutions; however, there is still an imbalance between different regions. Taking the “number of (assistant) physicians per 1,000 permanent residents in primary medical and health institutions” as an example, in the 23 integrated medical institutions, the index of those in developed regions with organized hierarchical diagnosis and treatment can exceed 0.5, which can be 0.1 or lower for those in underdeveloped regions with poor hierarchical diagnosis and treatment; similarly, as for the “proportion of (assistant) physicians assigned by hospitals to primary medical and health institutions,” in the 23 integrated medical institutions, the index of those in developed regions with organized hierarchical diagnosis and treatment can exceed 0.5, while there are barely practicing (assistant) physicians dispatched to grass-root medical institutions in underdeveloped regions with poor hierarchical diagnosis and treatment. “The number of equipment more than 10,000 yuan per 1,000 permanent residents in primary medical and health institutions” and “The number of beds per 1,000 permanent residents in primary medical and health institutions” are similar to “The number of (assistant) physicians per 1,000 permanent residents in primary medical and health institutions,” there are uneven and unbalanced conditions; The effective implementation of hierarchical diagnosis and treatment requires balanced development of integrated medical institutions in various regions. Therefore, government departments should take measures according to local conditions to increase the input of medical resources in grassroots medical institutions, promote a balanced allocation of hierarchical diagnosis and treatment resources, and realize a balanced development of hierarchical diagnosis and treatment.

#### 3.2.2. Communication and interaction

“Communication and interaction” consists of three indexes, namely “number of people per 100 health technicians in the hospitals to the primary medical institutions for guidance” and “number of new technologies and new projects performed by hospitals and primary health technicians per 100,” in which the “number of new technologies and new projects performed by hospitals and primary health technicians per 100” has the strongest impact on developing hierarchical diagnosis and treatment, followed by the “number of people per 100 health technicians in the hospitals to the primary medical institutions for guidance” and “number of people per 100 health technicians at the primary institutions studying in hospitals”. The “number of new technologies and new projects performed by hospitals and primary health technicians per 100” is a major manifestation of the degree of cooperation of medical personnel in superior and subordinate medical institutions, which can effectively improve the level of medical service of basic medical institutions. In the 27 tertiary indexes, its weight is 0.0471, ranking second; the “number of people per 100 health technicians in the hospitals to the primary medical institutions for guidance” is an important index reflecting the strengthening of training capacity for medical personnel in grass-root medical institutions, and also a major way to improve the capacity of medical service of grass-root medical institutions. In the 27 tertiary indexes, its weight is 0.0387, ranking the twelfth. The “number of people per 100 health technicians at the primary institutions studying in hospitals,” as part of the continuing education of health technicians at the grassroots level, is a major path to improve the capacity of medical service of these health technicians; In the 27 tertiary indices, its weight is 0.0272, ranking as 26th. Therefore, these two indexes have no strong impact on developing hierarchical diagnosis and treatment; the “number of people per 100 health technicians in the hospitals to the primary medical institutions for guidance” and “number of people per 100 health technicians at the primary institutions studying in hospitals” are slower to take effect, and no good results have been made; they are not the major indexes for evaluating the development of hierarchical diagnosis and treatment.

There is a great gap in the “number of people per 100 health technicians in the primary institutions studying in hospitals” and “number of people per 100 health technicians in hospitals to the primary medical institutions for guidance” among the 23 integrated medical institutions, some of which dispatch personnel of more than 100,000 person-time to provide guidance at the grassroots level and learn in hospitals, and some almost dispatch no personnel. Communication and interaction between superior and subordinate medical institutions are quite important for the construction of basic medical institutions and is also the basic requirement for the establishment of the hierarchical diagnosis and treatment system; therefore, it is necessary to take practical methods to increase the communication and interaction between these medical institutions according to the actual conditions of different types of medical institutions at different levels, optimize the allocation of resources for hierarchical diagnosis and treatment, and realize the sustainable development of hierarchical diagnosis and treatment.

### 3.3. Establishment of the hierarchical diagnosis and treatment management system

#### 3.3.1. Incentive compatibility

“Incentive compatibility” consists of four indexes, namely the “government financial investment policy”, “medical insurance incentive system”, “work quality assessment system of medical institutions” and “personnel salary system of medical institutions”, in which the “government financial investment policy” has the strongest impact on the development of hierarchical diagnosis and treatment, followed by “medical insurance incentive system” and “personnel salary system of medical institutions,” and the “work quality assessment system of medical institutions” has the least impact. Presently, medical institutions assume sole responsibility for their profits or losses under financial support. In a highly competitive medical environment, “government financial investment policy” can play an important incentive role in the implementation of hierarchical diagnosis and treatment by management personnel and medical personnel in an integrated medical service institution. Among the 26 tertiary indices, the weight of “government financial investment policy” is 0.0491, ranking the first and being the tertiary index with the highest impact. “medical insurance incentive system” is also important for the development of hierarchical diagnosis and treatment; Medical insurance, as an important measure of the new medical reform to promote hierarchical diagnosis and treatment and a powerful tool to regulate the medical behaviors of doctors and patients, can realize orderly medical treatment of patients, and regulate medical institutions to carry out diagnosis and treatment rationally, reduce medical costs and save medical expenses. As the main payer of medical expenses in China presently, the medical insurance incentive system has a key impact on the medical industry and its weight is 0.0449, ranking fifth in the 26 tertiary indexes. The weights of “personnel salary system of medical institutions” and the “work quality assessment system of medical institutions” are 0.0427 and 0.0300, ranking ninth and twenty-sixth, respectively.

The data from this study show that the overall level of construction of the medical insurance incentive system in Fujian Province is obviously insufficient, and the lack of continuous incentive policies leads to the lack of policy and financial support for the integration and coordination of hierarchical diagnosis and treatment. Any policy reform and innovation require a large amount of investment. Multiple integrated medical service institutions do not formulate corresponding government financial investment policies and do not invest special funds in the construction of hierarchical diagnosis and treatment (such as subsidies for sinking personnel, hanging personnel learning costs, and personnel performance subsidies). The existing incentive system construction in Fujian province cannot effectively support the development of hierarchical diagnosis and treatment. Most integrated health service institutions are coping with policy pressure. Individual integrated medical service institutions can find some ways to avoid some policies and obtain short-term benefits under the framework of hierarchical diagnosis and treatment.

#### 3.3.2. Division of labor and cooperation

Among the two indicators of “division of labor and cooperation,” the “resource division system” has a stronger impact on developing hierarchical diagnosis and treatment. Among the 27 tertiary indicators, the weight of the resource division system is 0.0454, ranking third; the weight of the resource integration system is 0.0257, ranking 27th. Hierarchical diagnosis and treatment emphasize the “primary diagnosis and two-way referral”. Its core is to require different levels of medical institutions to cooperate and share resources. The resource division system is the main measure to smooth two-way referral and the division and cooperation of different levels of medical institutions in the specific implementation process of hierarchical diagnosis and treatment. The resource integration system helps to optimize the allocation of medical resources, maximize the completion of medical services with limited medical resources, and improve people's health.

There is a big gap between the two indicators of the “resource integration system” and the “resource division system” in 23 integrated medical service institutions. Some integrated medical service institutions established a clear division and cooperation system between upper and lower medical institutions. Few integrated medical service institutions have almost no corresponding system construction. The construction of a “division of labor and cooperation system” is very important for the construction of primary medical institutions, and it is an important way to establish the hierarchical diagnosis and treatment system. Therefore, according to the actual situation of different levels and types of medical institutions, it is necessary to do a good job in the division of responsibilities, determine the diseases that are treated, improve the resource integration system, optimize the allocation of medical resources, and promote the high-quality development of hierarchical diagnosis and treatment.

### 3.4. Analysis of implementation effect factors for hierarchical diagnosis and treatment

#### 3.4.1. Triage effect

Among the five indicators of the “triage effect”, “utilization rate of inpatient beds in primary medical and health institutions” has the strongest impact on the development of hierarchical diagnosis and treatment, followed by the “proportion of outpatient and emergency visits in primary medical and health institutions”, the “proportion of up-down referrals” and the “proportion of level III and IV surgery in hospital,” and finally the “coincidence rate of main diagnosis in admission and discharge in hospital”. Among the 27 tertiary indicators, the weight of the “bed utilization rate in primary hospital” is 0.0478, ranking first. There is no doubt that the key to hierarchical diagnosis and treatment is strengthening the grass-roots level. One of the results of strong grassroots level is that there are more and more patients in primary medical institutions, and the number of hospitalized patients is gradually increasing. The use rate of primary hospital beds is further improved. The weight of “proportion of outpatient and emergency visits in primary medical and health institutions”, “proportion of up-down referrals,” “proportion of level III and IV surgery in hospital,” and “the coincidence rate of main diagnosis in hospitals” are 0.0429, 0.0381, 0.0369, and 0.0323, respectively. Among the 27 indicators, rank seventh, thirteenth, fourteenth, and twenty. Hierarchical diagnosis and treatment require division of labor and cooperation between higher and lower medical institutions. Primary medical institutions are primarily responsible for common diseases and chronic diseases, that is, the medical treatment task of most diseases. Therefore, the proportion of primary outpatient and emergency is also very important for developing hierarchical diagnosis and treatment, which is a symbol of the real realization of hierarchical diagnosis and treatment. For the superior hospital, its responsibility is to treat patients with difficult and critical diseases. The most important indicators are the proportion of third- and fourth-grade surgery or C- and D-type surgery, and the main diagnostic coincidence rate of hospital admission and discharge, which are important indicators to reflect the level of diagnosis and treatment and comprehensive strength of the superior hospital. The proportion of up-and-down referrals is the concentrated reflection of the close cooperation and the effect of up-and-down referrals.

The increase in the utilization rate of primary hospital beds can, in turn, encourage primary medical institutions to further increase investment to improve medical service levels, build celebrity, improve competitiveness, attract more patients to visit, and form a benign development cycle. In contrast, the low usage rate of hospital beds at the grassroot level is easy to form a vicious circle, resulting in the brain drain of grassroots medical institutions and the further shrinkage of grassroots medical institutions, which affects the formation of a hierarchical diagnosis and treatment system. Compared to the general high usage rate of hospital beds, the utilization rate of primary hospital beds is generally low. This study found that the usage rate of primary hospital beds in only seven samples exceeded 70% and the utilization rate of primary hospital beds in 27 samples was <10%, accounting for 39.13%. Based on this, it is still necessary to increase the input of medical resources in primary medical institutions, attract patients first diagnosis at the grassroots level, improve the usage rate of inpatient beds in primary medical institutions, form a benign development cycle, and provide the high-quality development of hierarchical diagnosis and treatment. The “proportion of outpatient and emergency visits in primary medical and health institutions” and the “proportion of up-down referrals” are the direct manifestation of the implementation effect of hierarchical diagnosis and treatment. The average proportion of primary outpatient and emergency visits in the sample data is only 32.83%, of which 19 samples are <20%; the proportion of referral visits in 56 samples was <10%, and some samples did not even make real referral visits. All these indicate that the triage effect of hierarchical diagnosis and treatment in Fujian province is not obvious enough, which is easy to cause a vicious circle. The enthusiasm of medical institutions and medical personnel at all levels to participate in hierarchical diagnosis and treatment will become increasingly weak, which seriously hinders the development of hierarchical diagnosis and treatment.

#### 3.4.2. Health benefits

Among the three indicators of “health benefits,” “contracted coverage rate of family doctors” has the strongest impact on developing hierarchical diagnosis and treatment, followed by the “chronic disease management rate” and “infant mortality”. Family doctor signing is the most important measure after the new medical reform. As the gatekeeper of residents' health, the family physician plays a vital role in the management and treatment of chronic diseases. Among the 27 three-level indicators, the weight of “contracted coverage rate of family doctors” is 0.0449, ranking fourth. The weight of “chronic disease management rate” and “infant mortality” are 0.0389 and 0.0369, respectively, and rank 11 and 15th among the 27 third-level indicators. The signing rate of family doctors is requiring the mature development of hierarchical diagnosis and treatment in the new period. It is also a policy measure that the health department attaches great importance to and promotes, and has also played a good effect. The weight of “chronic disease management rate” and “infant mortality” are relatively low, indicating that Fujian Province has done well in these areas and achieved certain results. The “chronic disease management rate” is the most important requirement and development goal in the context of healthy China, and it is also the embodiment of the hierarchical diagnosis and treatment goal. “Infant mortality” is the main influence indicator of average life expectancy in a region, and it is a measure indicator of health improvement in hierarchical diagnosis and treatment management.

In the sample data, the average coverage rate of family doctors is only 19.60%, and the coverage rate of family doctors in only four samples is more than 50%. As a hierarchical diagnosis and treatment policy implemented after the new medical reform, government departments have formulated family doctors' corresponding development requirements and implementation guidelines many times, which is the key to improving the service capacity of primary medical institutions, the key topic of chronic disease prevention and control, and the fundamental factor of whether hierarchical diagnosis and treatment can achieve high-quality development. Statistics from the WHO in 2017 show that chronic diseases cause 40 million deaths worldwide each year, accounting for 70% of total deaths. The number of deaths from chronic diseases is expected to increase to 55 million by 2030 (48). In China, patients with chronic diseases have exceeded 200 million, representing more than 20% of the total population (49). Research shows that in the population aged 60 and over in China, on average, each person has 1.61 kinds of chronic diseases and 44.44% of them have comorbidities, which makes medical and health expenditures in China increase significantly (50). The treatment of chronic diseases started late in China and was gradually developed in some areas in the 1980s (51), the average rate of chronic disease management in the sample is 75.90%, which is still low in general, and the level of management remains to be considered. In the context of healthy China, it is necessary to expand the coverage of chronic disease management, construct standardized chronic disease health management, strengthen the training of health management personnel, and continuously improve.

#### 3.4.3. Medical cost control

Among the three indicators of “medical cost control,” “change rate of self-payment burden per hospitalisation” has the strongest impact on developing hierarchical diagnosis and treatment, followed by “annual balance rate of revenue and expenditure,” and finally “balance rate of medical insurance fund”. The weight of “the change rate of hospitalization expenses per patient” is 0.0297, ranking twenty-second in 27 three-level indicators, the weight of annual income and expenditure is 0.0287, ranking twenty-third in 27 three-level indicators, the weight of the “medical insurance fund” is 0.0280, ranking twenty-fourth in 27 three-level indicators. The weights of the three indicators are arranged in the end, indicating that medical cost control is not the most critical indicator for evaluating the performance of hierarchical diagnosis and treatment in Fujian province presently. The change rate of hospitalization expenses per patient is relatively the most important indicator in “medical cost control,” which is related to the cost and burden of people's medical treatment. Reducing the cost and burden of people's medical treatment is a starting point for hierarchical diagnosis and treatment, and it is also one of the fundamental ways to solve “poverty caused by disease and return to poverty due to disease.”

In the sample data, only 24 samples “change rate of hospitalization expenses per patient” are negative, and 45 samples “hospitalization expenses per patient maintain an upward trend, some even more than 30%. Only 16 samples” annual revenue and expenditure ratios are positive, and most of the annual revenue and expenditure ratios are below 6%. The 53 samples of the “annual revenue and expenditure ratio” are in a deficit state, which must rely on financial subsidies to achieve development, lacking endogenous motivation to support high-quality development. In the increasingly fierce medical competition environment, the annual revenue and expenditure ratio is negative and can greatly affect the development of integrated medical services. In the sample data, only 11 samples “medical insurance fund knot rate” is negative, indicating that most of the samples of medical insurance funds can balance income and expenditure or a little surplus, reflecting the current integrated medical service institutions in Fujian Province do a good job in the use and management of medical insurance funds, can make good use of medical insurance funds to manage medical expenses, to achieve effective control of medical costs.

## 4. Conclusion

In this study, samples collected from 23 representative integrated medical institutions in nine Fujian cities from 2018 to 2020 are taken as subjects. A hierarchical diagnosis and treatment performance appraisal system is established based on the mechanism of research on the operation of hierarchical diagnosis and treatment. The weight of each evaluation index is determined with the CRITIC method, and the hierarchical diagnosis and treatment effects on 23 subjects are quantitatively evaluated by the Gray correlation method based on the weight of each index. Gray correlation of each year in Fujian has gradually increased. But there is still room for improvement. The government departments must improve the investment in medical resources with measures adjusted according to local conditions, promote a balanced allocation of resources for hierarchical diagnosis and treatment, increase communication and interaction between upper and lower medical institutions, and optimize the allocation of resources for hierarchical diagnosis and treatment.

The limitation of this study is that the survey sample is not nationwide.Future we consider expanding the sample to investigate integrated medical institutions in other provinces.

## Data availability statement

The raw data supporting the conclusions of this article will be made available by the authors, without undue reservation.

## Author contributions

QW came up with the original idea and model for this paper, calculated the data, and wrote the paper. ZZ adjust the overall format and content of the paper. XX was responsible for the guidance, review, and writing part of the paper. All authors contributed to the article and approved the submitted version.
